# Collaterally Sensitive β-Lactam Drugs as an Effective Therapy against the Pre-Existing Methicillin Resistant *Staphylococcus aureus* (MRSA) Biofilms

**DOI:** 10.3390/ph16050687

**Published:** 2023-05-02

**Authors:** Hamna Batool Hashmi, Muhammad Asad Farooq, Muhammad Hashim Khan, Abdulrahman Alshammari, Alanoud T. Aljasham, Sheikh Abdur Rashid, Nauman Rahim Khan, Irum Batool Hashmi, Muhammad Badar, Mohammad S. Mubarak

**Affiliations:** 1 Gomal Center of Biochemistry and Biotechnology, Gomal University, Dera Ismail Khan 29050, Pakistan; hamnahashmi27@gmail.com (H.B.H.); hashimkhan@gu.edu.pk (M.H.K.); 2 Shanghai Key Laboratory of Regulatory Biology, Institute of Biomedical Sciences, East China Normal University, Shanghai 200062, China; asadfarooq601@yahoo.com; 3 Department of Pharmacology and Toxicology, College of Pharmacy, King Saud University, P.O. Box 2455, Riyadh 11451, Saudi Arabia; abdalshammari@ksu.edu.sa (A.A.);; 4 Nanocarriers Research Laboratory, Gomal Centre of Pharmaceutical Sciences, Faculty of Pharmacy, Gomal University, Dera Ismail Khan 29050, Pakistan; 5 Department of Pharmacy, Kohat University of Science and Technology, Kohat 26000, Pakistan; 6 Department of Obstetrics and Gynecology, Gomal Medical College, Dera Ismail Khan 29050, Pakistan; 7 Department of Chemistry, The University of Jordan, Amma 11942, Jordan; 8 Department of Chemistry, Indiana University, Bloomington, IN 47405, USA

**Keywords:** methicillin-resistant *Staphylococcus aureus* (MRSA), antibiotic resistance, β-lactam antibiotics, biofilms, anti-bacterial activity

## Abstract

Methicillin-resistant *Staphylococcus aureus* (MRSA) is among the leading causes of nosocomial infections and forms biofilms, which are difficult to eradicate because of their increasing resistance to antimicrobial agents. This is especially true for pre-existing biofilms. The current study focused on evaluating the efficacy of three β-lactam drugs, meropenem, piperacillin, and tazobactam, alone and in combination against the MRSA biofilms. When used individually, none of the drugs exhibited significant antibacterial activity against MRSA in a planktonic state. At the same time, the combination of meropenem, piperacillin, and tazobactam showed a 41.7 and 41.3% reduction in planktonic bacterial cell growth, respectively. These drugs were further assessed for biofilm inhibition and removal. The combination of meropenem, piperacillin, and tazobactam caused 44.3% biofilm inhibition, while the rest of the combinations did not show any significant effects. Results also revealed that piperacillin and tazobactam exhibited the best synergy against the pre-formed biofilm of MRSA, with 46% removal. However, adding meropenem to the piperacillin and tazobactam combination showed a slightly reduced activity towards the pre-formed biofilm of MRSA and removed 38.7% of it. Although the mechanism of synergism is not fully understood, our findings suggest that these three β-lactam drugs can be used in combination as very effective therapeutic agents for the treatment of pre-existing MRSA biofilms. The in vivo experiments on the antibiofilm activity of these drugs will pave the way for applying such synergistic combinations to clinics.

## 1. Introduction

Methicillin-resistant *Staphylococcus aureus* (MRSA) is a round-shaped, non-motile gram-positive bacterium. Through natural selection or horizontal gene transfer, it has developed multiple resistances to β-lactam antibiotics, a class of broad-spectrum antibiotics comprised of carbapenems, penams, cephalosporins, and others [1]. β-Lactams act on the penicillin-binding protein (PBP) and thereby inhibit cell wall synthesis in actively dividing bacterial species [2]. However, all strains of MRSA are resistant to β-lactams because they have genetically acquired determinants such as *mecA* or *mecC,* which, when expressed, produce penicillin-binding protein 2a (PBP2A) or PBP2A’ that continue the cell wall synthesis in MRSA even in the presence of β-lactams [3]. Antibiotic resistance, biofilm-forming capacity, invasiveness, and toxin-mediated virulence are some characteristic features of MRSA, making it a highly dangerous pathogen [4,5]. In this respect, bacterial biofilms are responsible for approximately 80% of chronic and recurring microbial infections in the human body [6]. Biofilms are structurally complicated, self-produced, matrix-enclosed microbial communities that adhere to living or non-living substratum, interface, or each other [7]. One of the profound clinical complications of the biofilm mode of growth is the resistance of microbes to antimicrobial agents. In contrast to planktonic cells, bacteria in biofilm mode are more resistant to antimicrobial agents due to their slow metabolic rate and cell division, which are essential for antibiotic sensitivity [8].

Complete removal of biofilm is a challenging task that often leads to persistent infections. The biofilm mode of lifestyle, which gives broad-spectrum resistance against various antimicrobial agents, has yet to be completely understood [5]. In this regard, natural, synthetic, or mechanical products can inhibit or remove preformed biofilms. Bacterial resistance to antibiotics within the communities of biofilms results in recurrent and persistent infections [9]. Therefore, alternative approaches are required to control biofilm-associated infections. With the speed at which microorganisms are developing antibiotic resistance mechanisms, it is incredibly challenging to discover new antibiotics. One method is to reduce the microorganisms’ capacity for resistance and render them sensitive to antibiotics currently approved by the FDA [10,11]. Therefore, this profound clinical complication has directed researchers towards seeking urgent novel antimicrobial combination therapies that not only eradicate the biofilms but also inhibit resistance development.

**Figure 1:**
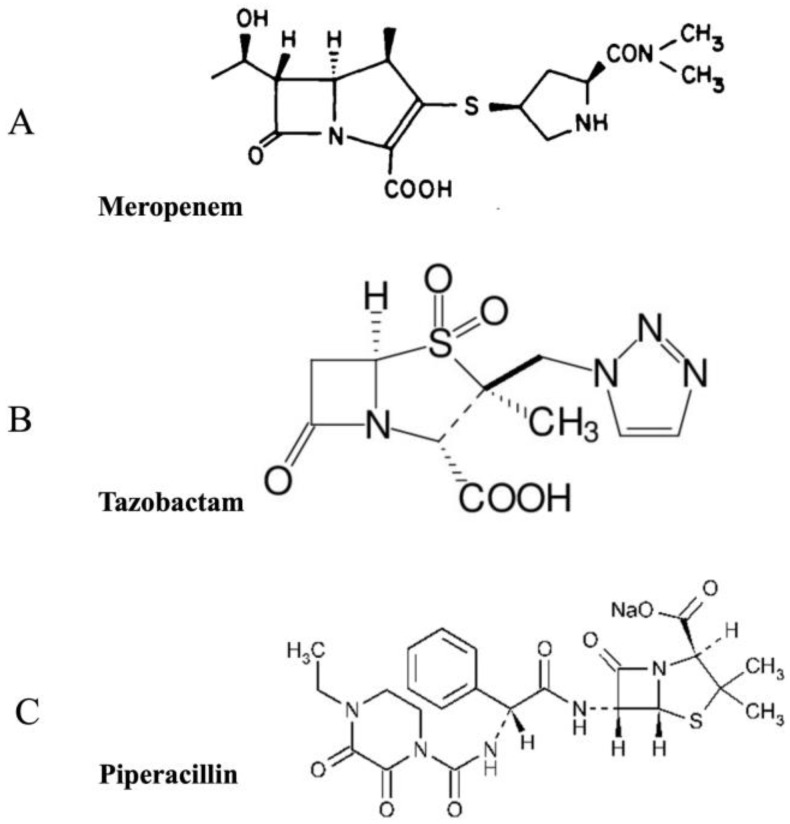
The chemical structures of (**A**) meropenem, (**B**) tazobactam, and (**C**) piperacillin.

A combination of three β-lactam drugs—meropenem, piperacillin, and tazobactam—has previously been identified [12] as a novel therapy for the treatment of MRSA. Meropenem is a broad-spectrum antibiotic with excellent in vitro bactericidal activity against nearly all clinically important aerobic and anaerobic bacterial pathogens. It is more active against *Pseudomonas aeruginosa*, all *Enterobactenaceae*, and *Haemoplulus influenzas* but is less active against *staphylococci* and *enterococci* [13]. It is clinically prescribed for bacterial meningitis, infection of skin and soft tissues, septicemia, pneumonia, cystic fibrosis, urinary tract infections, and obstetric and gynecological infections [14]. Piperacillin sodium inhibits the growth of gram-positive cocci (excluding penicillinase-producing *S. aureus*), gram-negative bacilli, and anaerobic pathogens such as *Clostridium difficile* and *Bacteroides fragilis*. It can inhibit many members of the *Enterobactenaceae,* such as *Pseudomonas* and *Klebsiella* spp. It is prescribed for urinary tract infection, intra-abdominal infection, inflammatory diseases of the pelvis, gynecological infections, septicemia, infections of the skin and soft tissues, uncomplicated gonococcal urethritis, and infections of bones and joints [15]. On the other hand, tazobactam has less intrinsic antibacterial activity. However, it can inhibit Richmond and Sykes types II, III, IV, and V β-lactamases, as well as staphylococcal penicillinase and extended-spectrum β-lactamases [16]. The chemical structures of the three drugs are shown in Figure 1. The three drugs reported here are approved by the Food and Drug Administration (FDA). Although obsolete now due to resistance development in MRSA against individual drugs, the triple drug combination, however, not only inhibits bacterial growth but also inhibits the evolution of resistance in multiple *S. aureus* strains [12], contrary to several other combinations that rather enhance resistance evolution in bacteria [17,18]. MRSA, like several other bacteria, becomes extremely resistant to antibacterial therapies during the biofilm mode of infection as compared to its planktonic phase. Based on the preceding discussion, the current study focuses on investigating the efficacy of these drugs against MRSA biofilms.

## 2. Results

### 2.1. The Antibacterial Activity of Piperacillin, Meropenem, and Tazobactam against MRSA in the Planktonic Phase

**Figure 2:**
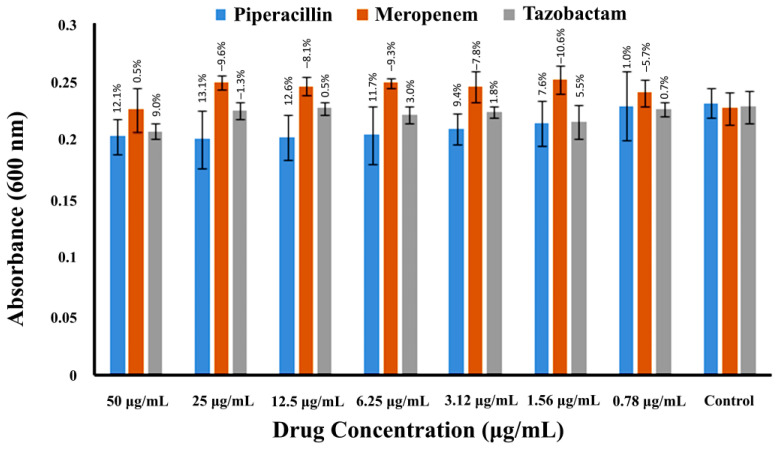
The antibacterial activity of piperacillin, meropenem, and tazobactam against MRSA in the planktonic phase. After 24 h of incubation of drugs with MRSA, the OD of planktonic bacterial cells was measured with the help of a spectrophotometer at 600 nm, and this value is presented as “absorbance (600 nm)” along the Y-axis. The X-axis shows the 2-fold serial dilution of drugs. Error bars indicate the ± standard deviation.

The antibacterial activity of piperacillin, meropenem, and tazobactam against planktonic bacterial growth was carried out using microtiter plates. The highest concentration of the drug used was 50 µg/mL in the 1st well, and after 2-fold serial dilution, the drug concentration was lowered to 0.78 µg/mL in the 7th well, and the eighth well served as the negative control. The antibacterial activities of three β-lactam drugs are shown in Figure 2 and Table 1. Results revealed a maximum of 13.1 and 9.0% reduction in bacterial cells for piperacillin and tazobactam, respectively, at 25 µg/mL. In contrast, meropenem showed the lowest antibacterial activity; it caused a 0.5% reduction in bacterial cell growth at the highest concentration of 50 µg/mL as compared to the control. The negative values in Figure 2 indicate that meropenem has a slightly antagonistic effect at all the concentrations studied except at the highest concentration (50 µg/mL). This was also true for tazobactam at 25 µg/mL; this effect, nevertheless, was statistically insignificant.

### 2.2. The Synergistic Antibacterial Activity of Combinations of Meropenem, Piperacillin, and Tazobactam against MRSA in the Planktonic Phase

**Figure 3:**
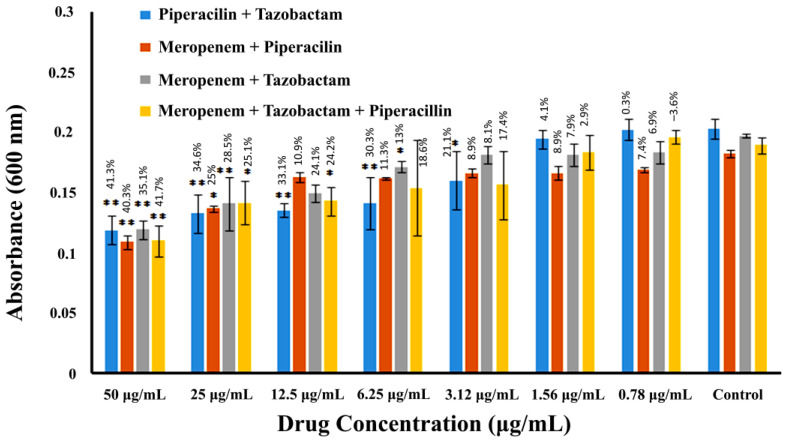
The antibacterial activity of combinations of β-lactam drugs against MRSA in the planktonic phase. After 24 h of incubation of drugs with MRSA, the OD of planktonic bacterial cells was measured with the aid of a spectrophotometer at 600 nm, and the resulting values are shown along the Y-axis as “absorbance (600 nm)”. The X-axis shows the 2-fold serial dilution of drugs in µg/mL. Percentage values over the bars represent the % reduction in bacterial growth compared to the control. Error bars indicate the ± standard deviation. Statistical significance is indicated by asterisks representing *p*-values (* *p* < 0.05). Two asterisks denote highly significant values (** *p* < 0.001).

A broth microdilution assay was used to test the efficacy of various drug combinations against MRSA in the planktonic phase. The highest concentration of the drug was 50 µg/mL; after 2-fold serial dilution, the concentration decreased to 0.78 µg/mL. The eighth well served as a negative control. The antibacterial activity of combinations of β-lactam drugs against MRSA in the planktonic phase is shown in Figure 3 and Table 1. All tested drugs showed synergistic effects in various combinations. The highly synergistic combinations were when all the drugs were used together, i.e., meropenem + piperacillin + tazobactam, or when piperacillin was combined with tazobactam.

The combination of meropenem + piperacillin + tazobactam reduced the bacterial cell density to 41.7% at 50 µg/mL concentration, proving highly significant (*p* < 0.001). The bacterial growth, however, increased towards higher drug dilutions, reaching a negative value (–3.6%) at the highest dilution, indicating a somewhat antagonistic effect, though non-significant. Similarly, the combination of piperacillin and tazobactam showed highly significant activity (*p* < 0.001) up to four dilutions and, at a 50 µg/mL concentration, caused a 41.3% inhibition of MRSA growth. Our findings also showed that the combination of piperacillin and meropenem exhibits highly significant antibacterial activity (*p* < 0.001); it caused a 40.3% reduction in bacterial cell growth at a 50 µg/mL concentration. In contrast, the combination of meropenem and tazobactam exhibited the least activity compared to the rest of the combinations. This combination caused a 35.1% reduction in MRSA cell growth at a concentration of 50 µg/mL. However, none of the drugs showed an antagonistic effect when combined with one or two other β-lactams as compared to when used alone.

### 2.3. MRSA Biofilm Inhibition by Piperacillin, Meropenem, and Tazobactam

**Figure 4:**
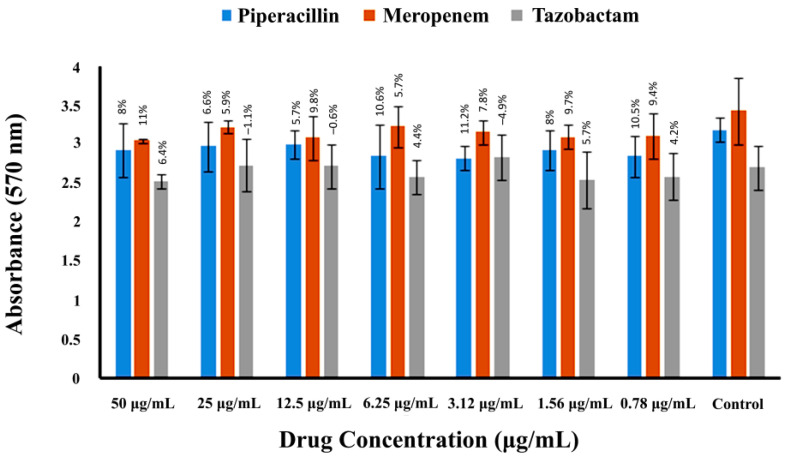
MRSA biofilm inhibition by meropenem, piperacillin, and tazobactam. The spectrophotometric absorbance measurements at 570 nm are presented along the Y-axis. The X-axis shows the two-fold serial dilution of drugs. Percentage values over the bars represent the % inhibition in the bacterial biofilm compared to the control. Error bars indicate the ± standard deviation.

To determine the extent to which three β-lactam drugs could inhibit the biofilm formation by MRSA, the MRSA strain and drugs were incubated in 24-well flat-bottom polystyrene multi-well plates as mentioned in the methods section. The biofilm inhibitory concentrations of meropenem, piperacillin, and tazobactam against MRSA are shown in Figure 4 and Table 1. Among the three drugs, meropenem, at its highest concentration (50 µg/mL), showed the maximum inhibition of biofilm formation, i.e., 11%. In contrast, piperacillin and tazobactam caused 8.0 and 6.4% inhibition of biofilm formation, respectively, at the highest drug concentration (50 µg/mL). None of the drugs caused substantial inhibition when compared to the control.

### 2.4. MRSA Biofilm Inhibition by Combinations of Meropenem, Piperacillin and Tazobactam

**Figure 5:**
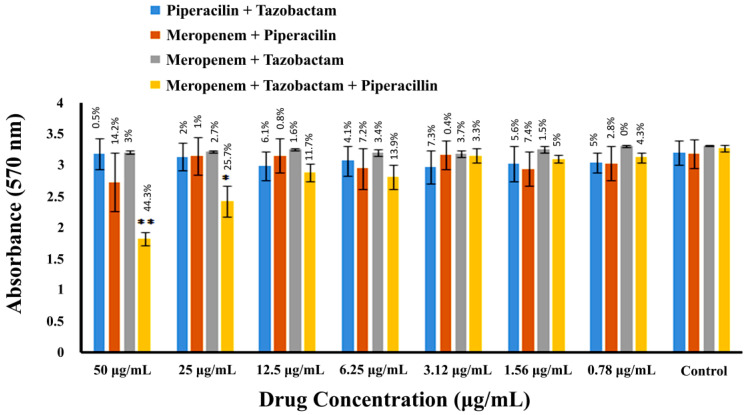
MRSA biofilm inhibition by combinations of β-lactam drugs. The spectrophotometric absorbance measurements at 570 nm are presented along the Y-axis. The X-axis shows the two-fold serial dilution of drugs in combinations. Percentage values over the bars represent the % inhibition in the bacterial biofilm compared to the control. Error bars indicate the ± standard deviation. Statistical significance is indicated by asterisks representing *p*-values (* *p* < 0.05), while the two asterisks denote highly significant values (** *p* < 0.001).

In the biofilm inhibitory concentration assay, MRSA cells were incubated with various combinations of drugs, and the resulting biofilms were stained and quantified, as mentioned in the experimental section. Displayed in Figure 5 and Table 1 are the biofilm inhibitory concentrations of combinations of meropenem, piperacillin, and tazobactam against MRSA. Results revealed that at the highest concentration of 50 µg/mL, a combination of meropenem, piperacillin, and tazobactam exhibited the highest significance (*p* < 0.001) and inhibited 44.3% of MRSA biofilm formation. The combination of meropenem and piperacillin inhibited 14.2% of MRSA biofilm formation. In contrast, the combination of meropenem and tazobactam showed a reduced effect compared to when they were used alone and inhibited only 3% of biofilm formation. Similarly, the combination of piperacillin and tazobactam showed minimum activity compared to the rest of the combinations and inhibited 0.5% MRSA biofilm formation at a 50 µg/mL concentration.

### 2.5. Removal of Pre-Formed MRSA Biofilms by Meropenem, Piperacillin, and Tazobactam

**Figure 6:**
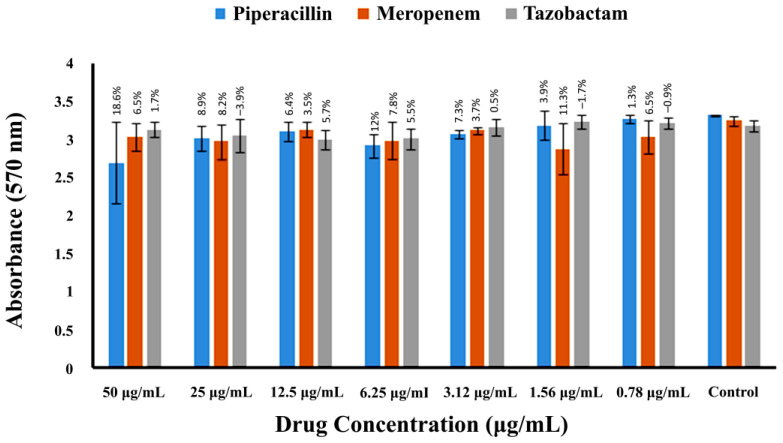
MRSA biofilm inhibition by meropenem, piperacillin, and tazobactam. The spectrophotometric absorbance measurements at 570 nm are presented along the Y-axis. The X-axis shows the two-fold serial dilution of drugs. Percentage values over the bars represent the % inhibition in the bacterial biofilm compared to the control. Error bars indicate the ± standard deviation.

The efficacy of three β-lactam drugs, meropenem, piperacillin, and tazobactam, was evaluated against the pre-formed biofilms of MRSA; the mature biofilms were stained and quantified after 24 h of drug incubation, as stated in the methods section. None of the drugs could effectively remove the biofilm compared to the control, as shown in Figure 6 and Table 1. Among the three drugs tested, piperacillin reduced 18.6% of biofilm at a 50 µg/mL concentration compared to meropenem and tazobactam, which showed a 6.5 and 1.7% reduction, respectively, at the same concentration.

### 2.6. Removal of Pre-Formed MRSA Biofilms by Combinations of Meropenem, Piperacillin, and Tazobactam

**Figure 7:**
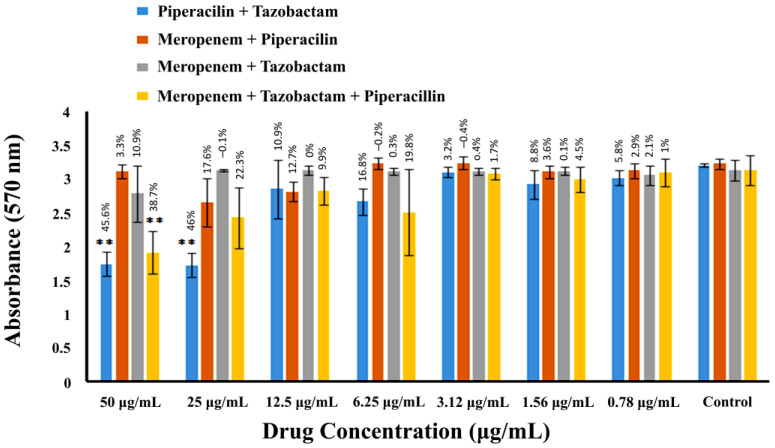
MRSA biofilm inhibition by combinations of β-lactam drugs. The spectrophotometric absorbance measurements at 570 nm are presented along the Y-axis. The X-axis shows the two-fold serial dilution of drugs in combinations. Percentage values over the bars represent the % inhibition in the bacterial biofilm compared to the control. Error bars indicate the ± standard deviation. The two asterisks denote highly significant values (** *p* < 0.001).

MRSA biofilm was formed in 24-well microtiter plates. After 72 h, the mature biofilm was incubated with different combinations of drugs for 24 h. After 24 h, staining and quantitation of biofilm were carried out. The in vitro biofilm reduction capability of combinations of β-lactam drugs against MRSA is shown in Figure 7 and Table 1. The combination of piperacillin and tazobactam proved highly synergistic and significant (*p* < 0.001); it reduced 46% of MRSA biofilm at 25 µg/mL. The second synergistic combination was that of piperacillin, tazobactam, and meropenem, which removed 38.7% of the pre-formed biofilm at a 50 µg/mL concentration. However, it also suggests that meropenem could have caused antagonistic effects on piperacillin because, when combined with piperacillin, it reduced 3.3% of the biofilm, which is less than piperacillin alone (18.6%) (see Figure 6). Similarly, when meropenem was combined with tazobactam, it reduced biofilm formation by 10.9%, showing a comparatively less synergistic effect.

## 3. Discussion

To the best of our knowledge, this study was the first to systematically examine the activity of meropenem, piperacillin, and tazobactam against MRSA biofilms. Our findings suggest that when used individually, none of the drugs exerts good antibacterial activity against MRSA in the planktonic phase compared to the control. Even the highest concentration of drugs (50 µg/mL) did not exhibit remarkable activity against MRSA. On the other hand, a highly synergetic combination was observed when the drugs (meropenem, piperacillin, and tazobactam) were combined against planktonic MRSA. The possible mechanism behind this synergistic triple antibacterial combination is interference with the synthesis of the bacterial cell wall. In this respect, meropenem and piperacillin can attack PBP1 and PBP2, respectively. Meropenem allosterically opens the active site of PBP2a, which makes it relatively easy for other β-lactams to attack it. Moreover, tazobactam suppresses the activity of β-lactamase. Hence, protection of piperacillin can be achieved, as β-lactamase has a high affinity for piperacillin [19].

Results from this investigation showed that none of the drugs substantially inhibited biofilm compared to the control. Because biofilm is a very resistant phenomenon, it has adopted different resistant mechanisms such as matrix, efflux pumps, and the presence of persisters, among others. Therefore, when drugs were used alone, no significant inhibition was achieved. Even against planktonic MRSA, none of the drugs could kill it alone. Thus, these drugs could not inhibit biofilm production because bacteria tend to attain 10 to 1000-fold higher resistance against antibiotics in the biofilm mode of growth than in the planktonic mode of development [20]. Within this context, research findings indicated that individual drugs do not kill pathogenic bacteria or inhibit biofilm production; therefore, the phenomenon of synergism or combination therapy evolved and became widely accepted [12,21]. When the three drugs were used in combination, appreciable biofilm inhibition was achieved. Therefore, it is assumed that the drugs might have interfered with the components of the matrix during its formation. eDNA in the matrix is one of the main components in biofilm formation. This eDNA also provides resistance to biofilms against various antimicrobial agents, such as aminoglycosides and cationic antimicrobial peptides, but not against β-lactams [22]. This could be the reason that the drugs interfered with the eDNA, hindering the formation of biofilm.

**Figure 8:**
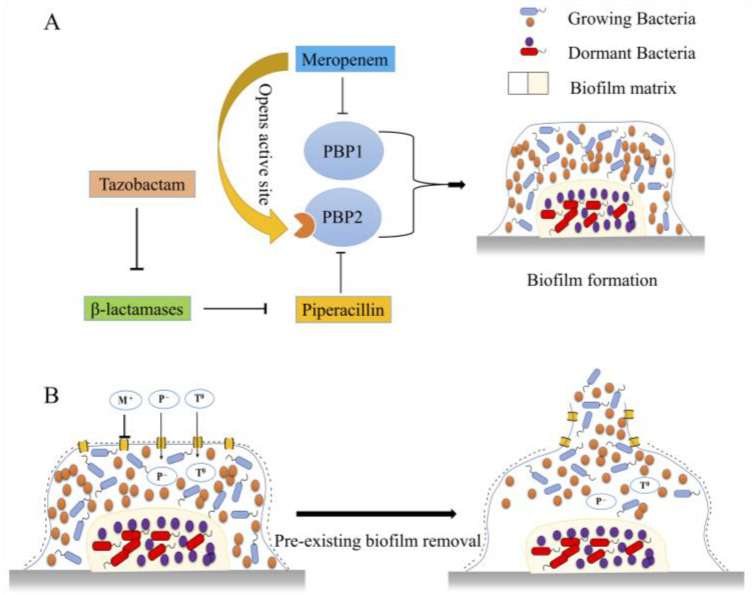
(**A**) Synergistic mechanism of drug combination in inhibiting biofilm formation. Meropenem targets PBP1, piperacillin targets PBP2, and tazobactam prevents the cleavage of piperacillin by β-lactamase. Meropenem also opens the active site on PBP2 for other β-lactam antibiotics. Both PBP1 and PBP2 promote biofilm formation. (**B**) Synergistic mechanism of pre-existing biofilm removal by drug combination. Biofilm has a negative charge, therefore, negatively charged piperacillin (P^−^) and neutral tazobactam (T^0^) can easily penetrate through the matrix, while positively charged meropenem (M^+^) is retarded by the negatively charged components of the matrix. Once inside the matrix, piperacillin can target PBP2 and tazobactam can protect it from β-lactamase degradation.

Research findings indicated that biofilm formation is impaired in *Escherichia coli* when PBP1b is deleted [23]. In contrast, impairment of PBP2a by *Acalypha wilkesiana* [24] prevented cell-surface attachment and biofilm formation in MRSA. In this regard, Tiwari et al. reported that meropenem and piperacillin target PBP1 and PBP2, respectively, while tazobactam prevents the cleavage of piperacillin by β-lactamase [25]. Hence, we assume that retardation of biofilm formation is accomplished by inhibition of PBP1 and PBP2, both involved in biofilm formation. However, this antibiofilm activity is reduced without tazobactam because piperacillin is targeted by β-lactamase, and PBP2 is not inhibited, which can lead to biofilm formation in the presence of meropenem and piperacillin. Additionally, we assume that tazobactam is the key player in the enhanced antibiofilm activity of these three drugs as it inhibits β-lactamase, which otherwise would degrade the β-lactam antibiotics (see Figure 8A).

Furthermore, our results also indicated that none of the three tested drugs could penetrate the biofilm when used individually to remove a pre-formed, mature, 72-hour-old, strong biofilm with well-developed resistance mechanisms. However, the combination of piperacillin and tazobactam proved highly synergistic. The possible synergistic mechanism for removing the pre-formed biofilm could be that the biofilm matrix has a negative charge; therefore, negatively charged piperacillin and neutral tazobactam can easily penetrate the matrix without being sequestered. Positively charged drugs are known to be affected by the negatively charged components of the matrix in the case of *P. aeruginosa,* while negatively charged drugs easily penetrate through it [26]. Once inside the biofilm, piperacillin can target PBP2, and tazobactam can protect it from β-lactamase degradation (see Figure 8B). This combination did not eliminate the biofilm because β-lactam drugs target the metabolically active cells at the periphery of the biofilm, while cells deeply rooted in the biofilm architecture often remain ineffective, which agrees with the existing data in the literature [27].

In addition to *mecA* or *mecC*, *S. aureus* also possesses various other resistance factors, like FmtA, which impart methicillin resistance to MRSA [28]. Researchers are, therefore, looking for novel molecules to deal with each of these resistance factors separately. Dalal et al. and Singh et al. have recently reported that various antibacterial molecules can bind to FmtA and can be considered potential candidates to encounter FmtA-mediated resistance in *S. aureus* [29,30]. Studies of MRSA resistance factors at the molecular level would greatly enhance our understanding of the drug interactions with its resistance factors and pave the way for designing effective treatments. Alternative approaches like phage therapy [31], nanoparticles [32], radiation therapy [33], and quorum sensing inhibition [34] have also been making grounds against MRSA resistance. Collateral synergy, repurposing of existing drugs, and the development of novel molecules that target different resistance factors in *S. aureus* are all essential to effectively combating this notorious pathogen [35].

## 4. Materials and Methods

### 4.1. Drugs, Stock Solutions, and MRSA

**Figure 9:**
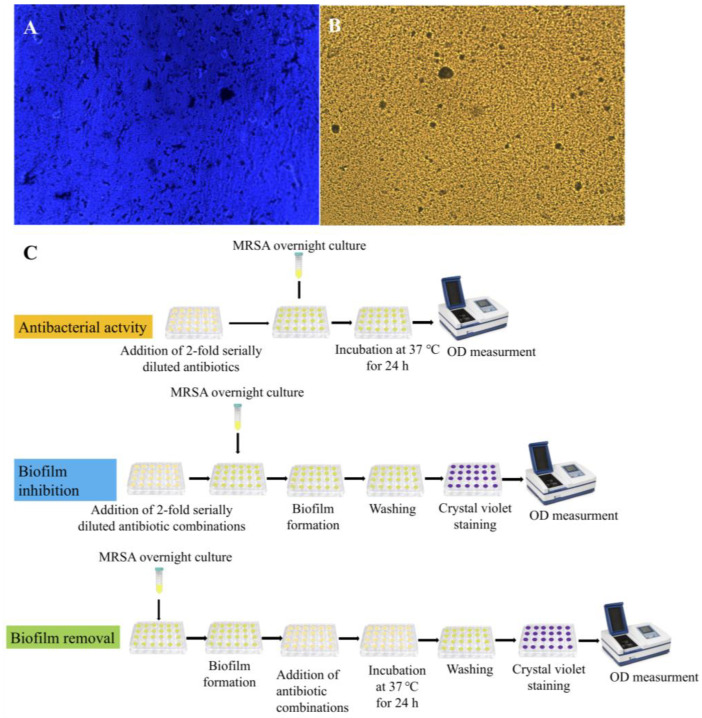
Light microscopy image of MRSA biofilm (**A**) stained with crystal violet; (**B**) unstained, Magnification = 200×; and (**C**) methodology flowchart.

Meropenem (CAS 96036-03-2), piperacillin salt (CAS 59703-84-3), and tazobactam (CAS 89786-04–9) were obtained from AK Scientific, Inc., Union City, CA, USA. The stock solution of drugs was prepared by adding 6.25 mg of each drug to 250 µL of dimethyl sulfoxide (DMSO) for a final stock concentration of 25 mg/mL. A potent biofilm-forming clinical isolate of methicillin-resistant *Staphylococcus aureus* (MRSA-S4) (Figure 9A,B) was obtained from the laboratory culture bank of the Gomal Center of Biochemistry and Biotechnology (GCBB). It was stored in glycerol at –20 °C. Prior to all assays, MRSA was grown overnight in tryptic soy broth (TSB) in an incubator at 37 °C. An overview of the methodology for the following experiments is shown in Figure 9C.

### 4.2. Determination of the Antibacterial Activity of Drugs

We have employed the broth microdilution method to determine the antibacterial activity of meropenem, piperacillin, and tazobactam, alone and in combinations (meropenem + piperacillin, meropenem + tazobactam, piperacillin + tazobactam, and meropenem + piperacillin + tazobactam) as per the Clinical and Laboratory Standards Institute (CLSI) guidelines. Briefly, 100 µL of TSB was added to the 2nd to 8th wells of the 96-well polystyrene microtiter plate in a row. Then, 100 µL of the drug solution alone (50 µg/mL) or in combination (1:1 mixture of each drug at 50 µg/mL concentration) prepared in TSB was added to the first well. Afterwards, 100 µL of the same drug solution was mixed with the TSB in the 2nd well, and a 2-fold serial dilution was prepared until the 7th well. The maximum concentration of the drugs thus used was 50 µg/mL, and the minimum drug concentration was 0.78 µg/mL. The eighth well served as a negative control, wherein 100 µL of TSB was added to mock the drug solution. This was followed by the addition of 10 µL from an overnight culture of MRSA with an OD_600_ of 0.5, prepared in TSB, to all wells. Optical density (OD) was measured at 600 nm with a spectrophotometer (HumaReader HS, Wiesbaden, Germany) at 0 h. The microtiter plate was incubated at 37 °C for 24 h. After 24 h of incubation, the microtiter plate was taken out of the incubator, and the ODs of all wells were measured at 600 nm. The antibacterial activity was determined by calculating the difference between the optical density of wells at 0 and 24 h.

### 4.3. Determination of Biofilm Inhibition

To determine the ability of drugs to inhibit the MRSA biofilms, 1 mL of each drug solution alone (50 µg/mL) or in combination (1:1 mixture of each drug with a 50 µg/mL concentration) prepared in TSB was 2-fold serially diluted in a 24-well flat-bottom polystyrene multiwell plate. The maximum concentration of the drugs thus used was 50 µg/mL, and the minimum drug concentration was 0.78 µg/mL. The 8th well served as a negative control whereas TSB was used to mock the addition of the drug. After serially diluting the drug, 100 µL of a static overnight culture of MRSA, grown in TSB with 2% glucose, having an OD_600_ of 0.5, was added to each well. After inoculation, the multiwell plate was placed in an incubator at 37 °C for 72 h. After 72 h, the multiwell plate was removed from the incubator and washed with distilled water 2–3 times, followed by staining with 1.5 mL of 0.1% crystal violet dye and incubation for 15 min. After 15 min, the plate was washed with distilled water 2–3 times to remove all the unbound dye. For the quantitation of biofilm, 1.5 mL of 30% acetic acid was added in all wells for 10–15 min to dissolve all the dye in the wells, and the OD of wells was measured at 570 nm.

### 4.4. Determination of Biofilm Removal

To determine the ability of the drugs to remove the preformed MRSA biofilms, the MRSA biofilms were first grown in a 24-well multiwell plate for 72 h. This was followed by the inoculation of 1 mL of each drug solution, alone (50 µg/mL) or in combination (1:1 mixture of each drug having a 50 µg/mL concentration), prepared in TSB to the first well. One milliliter of the same drug solution was mixed with equal amounts of TSB as per the standard procedure to prepare a 2-fold serial dilution in separate 15-mL falcons, and then added to the 2nd to 7th well. The maximum concentration of the drugs thus used was 50 µg/mL, and the minimum drug concentration was 0.78 µg/mL. The 8th well served as a negative control, which included additional TSB instead of drug solution. Great care was taken while inoculating the drug solution in wells so that the biofilm produced in them was not disturbed. The plate was placed in an incubator for the next 24 h at 37 °C. After 24 h, the plate was taken out of the incubator and washed and stained as previously described. Afterward, the biofilm was quantitated, and the readings were recorded for each well.

### 4.5. Statistical Evaluation

All experiments were performed in triplicate, and ANOVA-Single Factor was employed for comparisons between group values. Results were considered statistically highly significant when *p* < 0.001 and significant when *p* < 0.05.

## 5. Conclusions

In summary, results from this investigation showed that the combination of meropenem, piperacillin, and tazobactam enhanced the killing of planktonic MRSA cells and proved synergistic against MRSA biofilm formation. Moreover, the combinations also proved effective against the pre-formed biofilm of MRSA, which is often extremely difficult to eradicate. In addition, the current study highlights the potential of combination therapy with drugs for the effective eradication of pre-existing biofilms and lays the foundations for successful clinical treatment of persistent infections. Future studies may focus on studying different synergistic combinations of other classes of FDA-approved drugs to eradicate the biofilms of various other bacteria, such as *Pseudomonas aeruginosa*. A higher level of evidence must also be achieved via in vivo studies before such combination therapies can find their way to clinics.

## Figures and Tables

**Table 1:** Individual vs. combined activity of β-lactam drugs against MRSA.

MRSA Planktonic Growth Inhibition by Individual and Combinations of β-Lactam Drugs							
	50 μg/mL	25 μg/mL	12.5 μg/mL	6.25 μg/mL	3.12 μg/mL	1.56 μg/mL	0.78 μg/mL
Piperacillin	12.1%	13.1%	12.6%	11.7%	9.4%	7.6%	1.0%
Meropenem	0.5%	–9.6%	–8.1%	–9.3%	–7.8%	–10.6%	–5.7%
Tazobactam	9.0%	–1.3%	0.5%	0.3%	1.8%	5.5%	0.7%
Piperacillin + Tazobactam	41.3%	34.6%	33.1%	30.3%	21.1%	4.1%	0.3%
Meropenem + Piperacillin	40.3%	25%	10.9%	11.3%	8.9%	8.9%	7.4%
Meropenem + Tazobactam	35.1%	28.5%	24.1%	13%	8.1%	7.9%	6.9%
Meropenem + Tazobactam + Piperacillin	41.7%	25.1%	24.2%	18.6%	17.4%	2.9%	–3.6%
**MRSA Biofilm Inhibition by Individual and Combinations of β-Lactam Drugs**							
Piperacillin	8%	6.6%	5.7%	10.6%	11.2%	8%	10.5%
Meropenem	11%	5.9%	9.8%	5.7%	7.8%	9.7%	9.4%
Tazobactam	6.4%	–1.1%	–0.6%	4.4%	–4.9%	5.7%	4.2%
Piperacillin + Tazobactam	0.5%	2%	6.1%	4.1%	7.3%	5.6%	5%
Meropenem + Piperacillin	14.2%	1%	0.8%	7.2%	0.4%	7.4%	2.8%
Meropenem + Tazobactam	3%	2.7%	1.6%	3.4%	3.7%	1.5%	0%
Meropenem + Tazobactam + Piperacillin	44.3%	25.7%	11.7%	13.9%	3.3%	5%	4.3%
**MRSA Biofilm Reduction by Individual and Combinations of β-Lactam Drugs**							
Piperacillin	18.6%	8.9%	6.4%	12%	7.3%	3.9%	1.3%
Meropenem	6.5%	8.2%	3.5%	7.8%	3.7%	11.3%	6.5%
Tazobactam	1.7%	–3.9%	5.7%	5.5%	0.5%	–1.7%	–0.9%
Piperacillin + Tazobactam	45.6%	46%	10.9%	16.8%	3.2%	8.8%	5.8%
Meropenem + Piperacillin	3.3%	17.6%	12.7%	–0.2%	–0.4%	3.6%	2.9%
Meropenem + Tazobactam	10.9%	–0.1%	0%	0.3%	0.4%	0.1%	2.1%
Meropenem + Tazobactam + Piperacillin	38.7%	22.3%	9.9%	19.8%	1.7%	4.5%	1%

The symbol “–” indicates an antagonistic effect.

## Data Availability

Data is contained within the article.
